# Workplace Health Promotion in Italian University Employees: Effects on Body Composition and Mediterranean Diet Adherence

**DOI:** 10.3390/ijerph21081003

**Published:** 2024-07-30

**Authors:** Alessia Moroni, Margherita Micheletti Cremasco, Giorgio Gilli, Raffaella Degan

**Affiliations:** 1 Department of Life Sciences and Systems Biology, University of Torino, 10123 Torino, Italy; alessia.moroni@unito.it; 2 University Service Center in Hygiene and Sport Sciences (SUISM), University of Torino, Via Marenco 32, 10126 Torino, Italy; giorgio.gilli@unito.it (G.G.); raffaella.degan@unito.it (R.D.); 3 Department of Public Health and Pediatrics, University of Torino, 10126 Torino, Italy

**Keywords:** workplace health promotion, university, employees, body composition, anthropometry, Mediterranean diet

## Abstract

As Workplace Health Promotion is spreading among several working environments, the university context seems to be one of the best to apply primary prevention activities. Working in this direction, the University of Torino led the Wellness@Work for UniTo Project (W@W), with the aim of promoting employees’ health. Internal university professionals assessed body composition and adherence to the Mediterranean diet (MD), giving on-target advice for improving lifestyle. The aim of this paper is to evaluate the effectiveness of the W@W Project after a 4-month intervention period. This project was addressed to university employees, who could participate on a voluntary basis. Researchers assessed (T1) socio-demographic information and collected anthropometric variables. Body composition was evaluated through Classic and Specific Bioelectrical impedance Vector Analysis (BIVA). Adherence to the MD was assessed through the Medi-Lite questionnaire. After the assessments, participants were given 5-min counselling from internal professionals. After about 4 months, participants were supposed to undergo same assessments (T2). Overall, 479 workers joined the project, and of those, 246 came back for the T2 assessment. Globally, either anthropometric, body composition, or MD variables improved significantly after 4 months, both for male and female samples, suggesting how an easy-to-apply WHP intervention could help to improve workers’ health.

## 1. Introduction

Workplace Health Promotion (WHP) is defined as ‘the combined efforts of employers, employees and society to improve health and well-being of people at work’ [1]. Translating into practice, WHP is aimed at improving people’s health and awareness of long-lasting primary prevention benefits through interventions involving, for example, free health screenings (e.g., nutritional assessments), participated activities, courses, exhibitions, graphical messages, focus groups, and coaching, whether in presence or online. In order to reach the widest population possible, one of the most engaging environments is definitely the workplace. It provides an excellent setting for implementing health promotion treatments, particularly for those who are frequently challenging to get involved with. Employment conditions have a direct impact on employees’ physical, emotional, economic, and social well-being, with a consequent effect on the health of their families, communities, and the entire society [2]. Indeed, there are many studies in the literature that documented the effectiveness of different interventions applied in several companies, proving how simple behavior changes yielded significant results in employees’ health [3,4]. For example, a study conducted in Spain in a workplace environment yielded positive results in better adherence to a Mediterranean diet pattern in order to prevent and treat obesity. Researchers provided specific, targeted, and individually tailored feedback as well as an educational counseling session supplemented by a purpose-designed nutrition education manual, indicating how a brief dietetic intervention can have a valuable impact on diet-related obesity risk factors [5]. Also, in the healthcare workplace setting, a 12-month health promotion intervention was introduced to employees who had at least one cardiovascular risk factor [6]. The authors showed how blood pressure, waist/hip ratio (WHR) index, BMI, total cholesterol, triglyceride level, and blood glucose values were reduced, and the levels of physical activity and adherence to the Mediterranean diet had progressively increased. Moreover, an Italian study evaluating the nutritional profile and the environmental impact of meals consumed in a workplace canteen in the presence of a nudge (i.e., the Double Pyramid logo) combined with a web-based application promoting the Mediterranean diet (10-month period) showed how the choice of plant-based dishes, mostly those based on whole-grain cereals and legumes, and fish increased over time, while meat-based options decreased [7].

Following the above-mentioned notions, the university work context represents a distinctive environment for WHPPs. Firstly, the large and heterogenous workforce (e.g., professors, researchers, technicians, fellows, etc.) can preciously contribute to the community’s wellness and thus to the internal implementation of interventions. Indeed, universities provide a variety of facilities and resources in addition to having an organized communication system that enables the delivery of communications, education, and skills. Moreover, the university institution plays an important role as an example for the community, for the applicability of good practices in working conditions, and for the promotion of worker relations [3,8,9], whose effectiveness can directly be spread to the whole students body.

In regard to interventions applied in the university context, the literature is lacking, especially in concern to convergent and multidisciplinary projects involving internal and different professionals and health fields (physicians, kinesiologists, nutritionists, psychologists, etc.). It has indeed been highlighted the importance of favoring multicomponent and multilevel interventions, investing a great deal of internal resources, and supporting communication networks. Despite the lack of extensive evidence, the benefits of even one-field (e.g., physical activity) intervention in such a context seemed to have a positive impact on employees’ well-being. Indeed, a recent scoping review led by the authors [3] and conducted on 12 studies in the university context documented that, regardless of the suggested activities and the examined outcomes, the majority of the studies found favorable findings for the overall employees’ wellness. It was, however, difficult, due to the limited number of studies and their extensive heterogeneity, to define and draw conclusions that could be helpful in implementing future guidelines for such interventions.

Moreover, the results revealed a knowledge gap in the research literature on nutrition education in conjunction with physical activity (PA), which appears to be the most successful intervention [4,10,11]. As stated in the introduction of the abovementioned scoping review, a proposition in this direction is indicated in the University of Torino’s (UniTo) 2021–2026 Strategic Plan, whose initiatives include the Wellness@Work for UniTo (W@W) Project for staff members (UniTo Piano Strategico, 2021–2026, par.1.3.2; Geuna et al., 2022). This project, promoted by the Rector of the University of Torino and internally funded by the General Direction, was conducted by a multidisciplinary group: the University Service Center in Hygiene and Sport Sciences (SUISM), the Laboratory of Anthropology, Anthropometry, and Ergonomics, the Functional and Neuromuscular Group, and the Department of Sciences of Public Health and Pediatrics, all in collaboration with the Direzione Sviluppo Organizzativo e delle Risorse Umane e Supporto Istituzionale ai Dipartimenti and Area Formazione. W@W was developed with the aim of promoting employees’ health by assessing their physical capacities, body composition, or adherence to the MD through rapid, non-invasive, and reliable tests evaluated by qualified professionals at the University of Torino. Such evaluations/information considerably contribute to the promotion of wellness/well-being and to the improvement of health by avoiding or reducing the outbreak, onset, and development of diseases or adverse events (primary prevention). In particular, the project also allowed researchers to identify problems of overweight and incorrect diet at an early stage in order to make interventions and possibly counteract their progression.

In light of the abovementioned information, the aim of this paper is to evaluate the effectiveness of the W@W for UniTo project with regard to body composition and adherence to the MD after a 4-month intervention period.

## 2. Materials and Methods

Data were collected in the context of the Wellness@Work for UniTo (W@W) Project from October 2020 to December 2021. The project and study design were approved by the Bioethics Committee of the University of Torino (17 December 2019, prot. N. 20290), and the data were collected according to the Declaration of Helsinki.

### 2.1. Sample and Study Design

The sample was composed of administrative employees, professors, researchers, PhD students, and fellows who could, on a voluntary basis, book a 40-min appointment with the project’s staff during working hours. The recruitment involved an on-target mail with the possibility of booking the appointment through a dedicated webpage. Employees were free to choose their preferred/most suitable day and hour using a digital calendar that showed the available slots.

**Figure 1:**
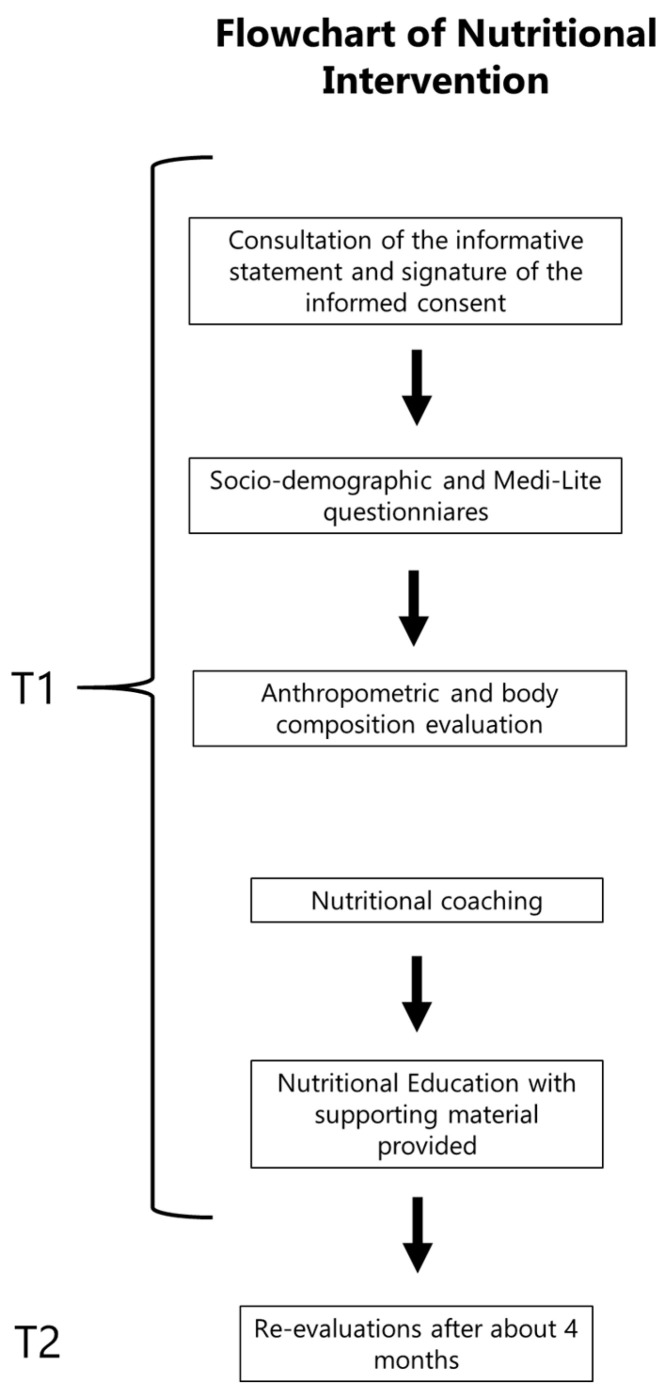
Nutritional intervention flowchart.

The study design included a baseline appointment (T1) ending with ad hoc advice by internal professionals, for improving body composition and adherence to the MD, and a second one after about 4 months (T2) in order to detect possible changes and assess the efficacy of the intervention. The entire process can be visualized in the flowchart below (Figure 1).

After consulting the informative statement and signing the informed consent, assessments were conducted as follows:

1. Filling an ad hoc socio-demographic questionnaire with information concerning birth year, civil status, educational level, sex at birth, profession, and liquid and soft drinks consumption.
2. Completing the Medi-Lite questionnaire [12,13], which assesses adherence to the MD. This questionnaire is available online (www.medi-lite.com), and it is composed of simple questions, allowing the user to define consumption in terms of daily and/or weekly consumption of 9 food groups identified and described in studies that investigated the association between adherence to the Mediterranean diet and health status. The score ranges between 0 (lowest adherence) and 18 (full adherence). Scores between 0 and 5 indicated scarce adherence, while between 6 and 12 point out medium adherence. Optimal adherence results were noted with scores between 13 and 18.
3. Anthropometric measurements and body composition assessment.
  a. Anthropometric variables were collected following the international standards and literature guidelines. Researchers measured weight and stature as well as waist, calf, and arm circumferences [14,15].
  b. In order to assess body composition, researchers measured bioelectrical variables, resistance (Rz) and reactance (Xc), using the phase-sensitive BIA 101 New Edition analyzer (50 kHz, 400 μA) and mono-use electrodes Biatrodes (by Akern Srl., Pontassieve, Florence, Italy), following the international standard procedures published by the National Institute of Health (NIH) [16]. Participants removed metal objects and were measured wearing comfort clothes (e.g., sport suit) with an empty bladder and skin free of oils or body lotions. Volunteers laid on their backs on a medical non-conductive surface (bed), and bioelectrical tissue values were assessed on the right hemisoma between the ipsilateral wrist and anklebone prominences (metatarsus/metacarpus region). The distance between the electrode pairs was 5 cm. Body composition quantitative estimates (i.e., Fat Mass, Fat Free Mass, etc.) were obtained through the Bodygram Dashboard^®^ v.3.0 (Akern Srl), while classic and specific BIVA corrections were performed according to Piccoli et al. (1994) [17], Buffa et al. (2013) [18], and Marini et al. (2013) [19], as better described in the data analysis paragraph.
4. Counselling the participants: five-minute primary prevention advice based on results of either the Medi-Lite or body composition outputs. Health professionals gave specific advice regarding specific food group integration or reduction according to their MD adherence, trying to guide them to behavioral changes towards healthier eating. Moreover, they answered all the questions from the participants and suggested that they periodically self-monitor the Medi-Lite score at home during the following month. The activity was included in the employee’s training file and accounted for 4 h of training, in which they also had to consult informational and educational material they were provided within an online platform. The nutritional training included a 2-h seminar in which researchers explained healthy eating habits along with Mediterranean diet patterns and beneficial effects. Moreover, the Italian Guidelines for a healthy nutrition [20] and links to the “healthy nutrition” government pages were included. All the participants had to consult the resources in order to complete the project.
5. Follow up: Re-evaluation after about 4 months.

### 2.2. Data Analysis

Data analyses were performed using IBM SPSS ver. 28 (IBM Corp., Armonk, NY, USA). Researchers used the paired *t*-test in order to investigate differences in anthropometric, body composition, and MD adherence values between T1 and T2.

The Body Mass Index (BMI) was calculated as kg/m^2^ in order to evaluate participants’ ponderal status categories: underweight (values ≤ 18.4 kg/m^2^), normoweight (values between 18.5 and 24.9 kg/m^2^, overweight (values between 25.0 and 29.9 kg/m^2^), and obese (values ≥ 30 kg/m^2^) [21]. McNemar’s test was used to analyze differences in ponderal status between T1 and T2.

For waist circumference cut-offs, the researchers used those introduced by the WHO (2011): adequate values for females are <80 cm, while for males <94 cm.

As stated above, bioelectrical raw data (resistance, R, and reactance, Xc) were analyzed using the Bodygram Dashboard^®^ and its equations cut-offs in order to estimate the following body compartments: Fat Mass (FM), Fat Free Mass (FFM), Muscle Mass (MM), Skeletal Muscle Mass (SMM), Appendicular Skeletal Muscle Mass (ASMM), Fat Mass Index (FMI), and Fat-Free Mass Index (FFMI).

The analysis of bioelectrical variables was then performed according to classic BIVA [17], correcting variables for stature (R/H, Xc/H, and Z/H) and according to specific BIVA [18,19], which corrects for both stature and cross-sectional areas (A/L), where A is estimated as 0.45 mid-arm area + 0.10 waist area + 0.45 calf area, yielding specific resistance, reactance, and impedance (Rsp, Xcsp, and Zsp, respectively). Arm, waist, and calf areas were estimated by the formula C2/4π, where C (m) is the circumference of the respective segment. Phase angle was calculated as follows: φ = arctan Xc/R·(180/π), and is not affected by the correction described for classic and specific BIVA. In both BIVA approaches, as first shown by Piccoli et al. [17] for classic BIVA, impedance can be considered a vector whose interindividual variability is plotted in a Cartesian plane, defined by a bivariate distribution of its components (R/H and Xc/H or Rsp and Zsp). Three tolerance ellipses are considered, covering areas including 50%, 75%, and 95% of the whole reference population. Intergroup comparisons were assessed using confidence ellipses. For classic BIVA, vectors moving parallel to the major axes indicate changes in tissue hydration (dehydration corresponds to long vectors, and hyperhydration is linked to short vectors), while vectors moving parallel to the minor axes indicate changes in the phase angle (higher values on the left), which means modifications in the cellular mass. For specific BIVA, vectors displacing parallel to the major axes indicate modifications in Fat Mass percentage (FM%) (more FM% corresponds to long vectors), and vectors moving parallel to the minor axes yield the same information as classic BIVA.

BIVA software and specific BIVA software (www.specificbiva.com, accessed on 10 June 2024) were used to draw tolerance ellipses and confidence ellipses and perform independent and paired T^2^ Hotelling tests. Statistical significance was accepted as *p* < 0.05.

## 3. Results

Overall, 479 employees of the UniTo joined the project at T1, but only 246 of these (aged 47.74 ± 9.34) came back after about four months (T2) and undertook the same assessments again. This study’s sample was composed of 165 females (aged 48.33 ± 9.04; 145 administrative or data technicians and 45 academic employees, including professors, researchers, PhD students, and fellows) and 81 males (aged 46.54 ± 9.88; 46 administrative or data technicians and 35 academic employees).

### 3.1. Baseline Sample

Descriptive statistics of the female and male samples are presented in Table 1 and Table 2, respectively, including anthropometric estimates as well as classic and specific bioelectrical variables. The ponderal status of the sample in terms of BMI and waist circumference is illustrated in Figures 3 and 6, respectively.

According to the World Health Organization (1997) [22], as shown in Figure 6, more than 60% of the baseline sample were normoweight, while almost 35% of participants were in conditions of overweight or obesity. Less than 3% were underweight. Dividing also by gender, females resulted in a major percentage of normoweight participants with respect to males (66.7% vs. 51.9%, respectively): indeed, males showed a consistent percentage of overweight or obese employees (~47%), while females in such conditions were 30.3%. For both sexes, underweight participants were less than 5%.

**Figure 2:**
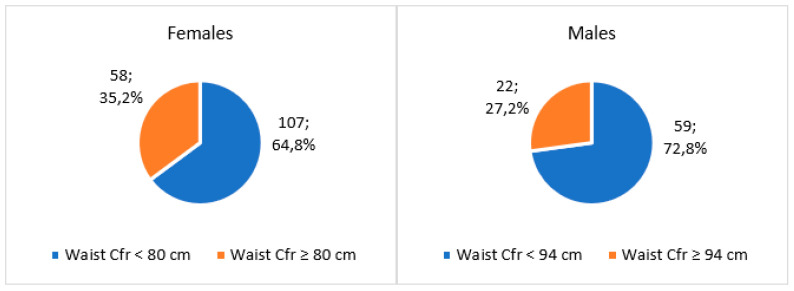
Ponderal condition of the baseline sample (*n* = 246) in terms of waist circumference divided by gender.

Considering the waist circumference (Figure 2), females showed a higher percentage (35.2%) of participants with values over the cut-offs with respect to males (27.2%), although for both samples, the majority of employees resulted within the adequate values.

**Figure 3:**
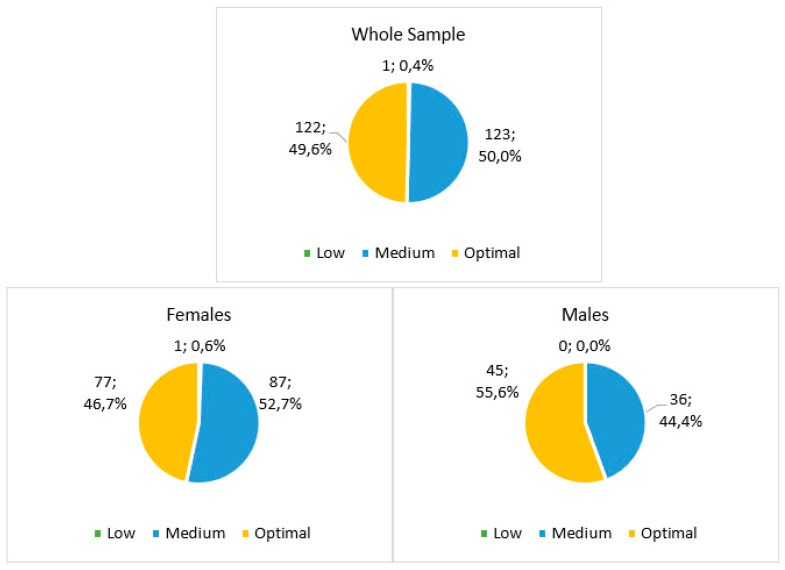
The baseline Medi-Lite score of the sample (*n* = 246) divided by sex.

Regarding MD adherence, half of the baseline sample reported medium adherence to the MD, while the other half resulted in the optimal score range. Dividing by gender, males showed a higher percentage of participants in the optimal range (more than 50%), and both sexes showed the same trend for the whole sample (Figure 3).

Generally, most of the baseline sample, in terms of BMI and waist circumference, resulted in normoweight and ponderal health, as well as being quite healthy in terms of eating habits.

### 3.2. Anthropometric and Body Composition Changes between T1 and T2

Regarding the female sample (*n* = 165, Table 1), significant differences were found for weight, BMI (*p* < 0.05), waist (*p* < 0.001) and hip circumferences (*p* < 0.01), FM, FMI, and FM% (*p* < 0.001), for which values decreased over the 4-month period, while FFM% significantly increased (*p* < 0.001). Classic BIVA variables decreased over time (R/H, Xc/H, and Z/H; *p* < 0.05), highlighting hydration improvement. Specific BIVA variables (Rsp, Xcsp, and Zsp; *p* < 0.001) significantly decreased at T2, indicating a notable decrease in FM%.

**Figure 4:**
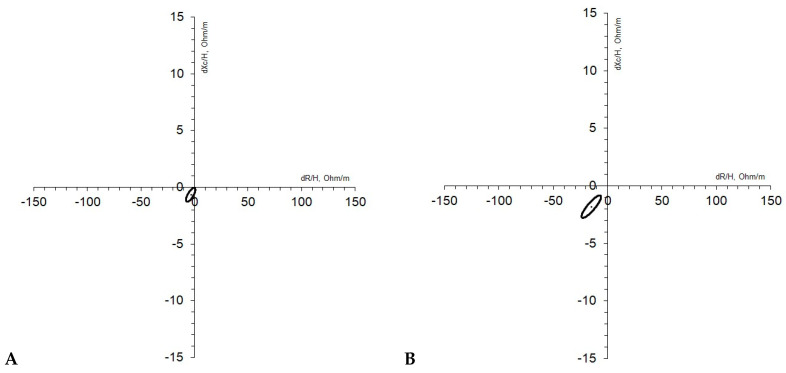
(**A**,**B**) Female sample (*n* = 165). Classic (**A**) and specific (**B**) results of confidence ellipses carried out with paired T2 Hotelling for classic BIVA (**A**) between T1 and T2 and results of confidence ellipses carried out with paired T2 Hotelling for specific BIVA between T1 and T2.

Regarding qualitative longitudinal vector analysis, classic BIVA (Figure 4A) highlighted a significant increase in hydration between T1 and T2, as the ellipse did not overlap with the origin of axes and the mean vector shortened its length (T^2^ = 7.0; *p* < 0.001). The specific BIVA ellipse (Figure 4B) distinctly did not overlap with the origin of axes, and such a result confirmed a significant decrease in FM% over the intervention period (T^2^ = 20.8; *p* < 0.001).

With regard to the male sample (*n* = 81, Table 2), significant decreases were observed for waist and hip circumferences, FM, FM%, and FMI (*p* < 0.05), while FFM, FFM%, and FFMI resulted significantly increased at T2 (*p* < 0.05). For classic BIVA variables, significant decreases were found only for R/H and Z/H, indicating a slight increase in hydration, as Xc/H did not reach significance but its *p*-value was equal to 0.05. Specific BIVA variables’ decrease (Rsp, Xcsp, Zsp, *p* < 0.01) highlighted a significant decrement in qualitative FM% in the 4-month period of intervention.

**Figure 5:**
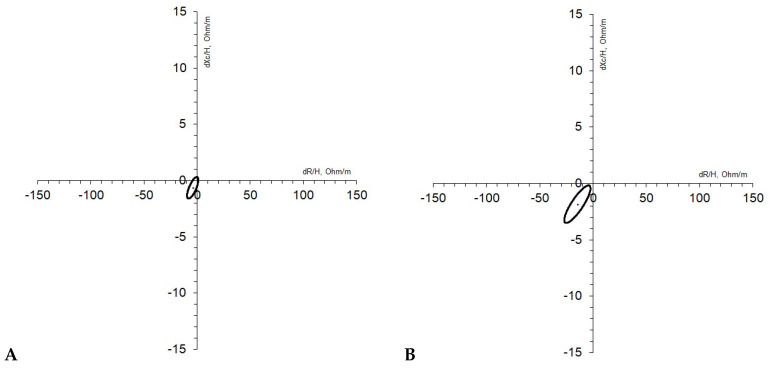
(**A**,**B**) Male sample (*n* = 81). Classic (**A**) and specific (**B**) results of confidence ellipses carried out with paired T2 Hotelling for classic BIVA (**A**) between T1 and T2 and results of confidence ellipses carried out with paired T2 Hotelling for specific BIVA between T1 and T2.

Classic BIVA longitudinal vector analysis (Figure 5A) did not show any significant modifications in hydration, while specific BIVA ellipses (Figure 5B) distinctly detached from the origin of the axes, pointing out a significant decrease in FM% between T1 and T2 (T2 = 9.2; *p* < 0.001).

### 3.3. Ponderal Status between T1 and T2

**Figure 6:**
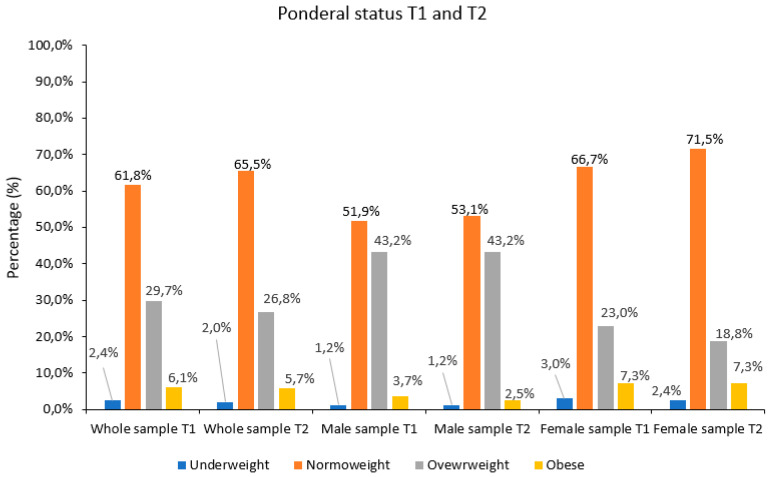
Ponderal condition of the sample (*n* = 246) in terms of BMI divided by gender at T1 and T2.

After 4 months of intervention, the percentage of individuals in the overweight and obese categories decreased for the whole sample, and improvements were also detected for both sexes (Figure 6), but all the identified changes were not significant (McNemar test). Males’ ponderal condition improved slightly at T2, remaining stable and very high in overweight individuals, while the percentage of obese individuals decreased. In the female sample, the percentage of overweight individuals decreased (reaching under 20%), thus resulting in over 70% of the sample being in a normoweight condition at T2.

### 3.4. Medi-Lite Score Changes between T1 and T2

Globally, the whole sample improved their dietary habits, as their Medi-Lite score increased significantly (from 12.35 ± 2.22 to 12.93 ± 2.11; delta mean 0.56 ± 1.97; t = 4.40; *p* < 0.001), remaining in the medium adherence range, despite the mean T2 score nearly reaching the optimum range, as the value was extremely close to 13.00.

The female sample significantly improved its score (*p* < 0.001), from 12.26 ± 2.13 to 12.85 ± 2.00, although it remained in the medium range of adherence. The male sample also improved its score (*p* < 0.05) and reached the optimal range of adherence (from 12.61 ± 2.35 to 13.10 ± 2.31).

## 4. Discussion

In the context of the W@W Project, which was addressed to University of Torino employees, the aim of this work was to assess and evaluate body composition and adherence to the MD in order to understand the efficacy of the activities and advice provided by internal professionals. Our results demonstrated how, at a follow-up after only 4 months, an overall improvement in body composition and a better adherence to the MD were observed. For the 246 employees who came back for the follow-up, the results showed that females tended to improve their body composition status more than men, as they lost weight along with FM (quantitatively and qualitatively) and conversely incremented FFM. Regarding BMI, it is notable to underline how females’ ponderal status improved more with respect to males, as the percentage of normoweight increased by 4.8%, reaching 71.5% after only 4 months. Females also enhanced their hydration status, as shown by classic BIVA results. Although males did not lose weight, their body composition significantly improved, as they still lost FM (quantitatively and qualitatively) and incremented FFM. Despite that, the increase in the percentage of normoweight individuals was only 1.2% after the intervention.

Concerning adherence to the MD, males showed a better trend with respect to females, as the baseline values of the Medi-Lite questionnaire highlighted a slightly higher adherence to the MD in males than females. Such a difference was still maintained at T2: in fact, neither females or males improved their adherence to the MD, but males reached the optimum range of adherence while females remained in the medium adherence range (despite higher statistical significance than males: females, *p* < 0.001; males *p* < 0.05). Dinu and collaborators [23] stated how the relationship between MD adherence and sex has not yet been well established, despite lots of studies reporting significant but divergent results (no differences depending on sex [24,25]; lower adherence in females [26,27,28] in the Spanish and Italian populations; and higher adherence in Italian females [29]). Although it was demonstrated that females are more aware of healthy eating choices for themselves and their families [25,26,27,28,29,30], such differences remain to be solved [23].

The convergent improvement of both body composition indicators as well as dietary habits was a great sign of the effectiveness of combined interventions of WHP, as nutrition and PA advice as well as their monitoring could not only improve worker’s health but also prevent NCDs. Indeed, the offer of internal qualified professionals’ competences to all university employees, along with their cooperation, showed promising preliminary results, in line with other interventions applied in other contexts.

As mentioned in the Introduction section, the literature concerning nutrition combined programs in universities is lacking, but it was well demonstrated how such programs resulted in effectiveness in other working realities, such as companies and institutions. Indeed, in the review of reviews conducted by Proper and van Oostrom [4], who analyzed 23 papers on several work environments, weight-related outcomes demonstrated strong evidence for favorable effects of WHP interventions, especially targeting physical activity and/or diet. In Italy, Lazzeri and colleagues [31] led a 1-year intervention on employees of the Siena University Hospital (Azienda Ospedaliera Universitaria Senese: AOUS), combining good practices of nutrition (consumption of fruit and vegetables) and physical activity. Despite the fact that there were no changes after the program period, the employees’ perceptions were satisfactory, and the authors stated that “the monitoring of risk factors needs to be continued over a longer period, in order to detect the appearance of the expected changes in the long term”. Cremaschini and collaborators [32] led a study in the companies’ setting of different kinds in the Province of Bergamo, finding positive effects (after 12 months) related to the consumption of healthier foods (fruit and vegetables), which were more evident in males and white-collar workers.

It is thus really difficult to compare our study with similar interventions, as either the setting or the intervention design and strategy are not likely to be comparable. However, despite such issues, implementing a multicomponent intervention in a favorable environment such as universities can thus lead to promising results and could be also spread to the student body in order to reach the most population possible, make people aware of their status, and potentially early detect risk factors for health. Indeed, we believe that the positive results are due to the multidisciplinary nature of the program, involving both nutrition and body composition’s improvement advice, as also shown by Rapisarda et al. (2021) [6], whose intervention involved multicomponent activities, yielding positive results. In order to improve the intervention, participants should be monitored more often, to provide more advice once they return, and to maintain a healthy lifestyle.

Moreover, it is important to highlight that, considering how the majority of the baseline sample resulted in being normoweight, from our results emerged an improvement in body composition and lifestyle habits in a context where results tend to take more time, as also described by Lazzeri et al. (2019) [31]. Indeed, when the sample is mostly composed of overweight or obese individuals, results are more likely to emerge in a short period of time, while it is not so easy to work on normoweight people and thus on primary prevention [33]. In this case, the aim was not to fight obesity but to promote health and lifestyle in order to prevent people from contracting NCDs. The attention of the professionals to the participants in terms of giving ad hoc advice and making them aware of their conditions could thus improve, even in just 4 months, the number of normoweight people and their eating habits, resulting in a healthier working force.

### Limitations

Our study presented some limitations: Firstly, due to the restricted number of participants, we could not generalize such results as representative of the entire employee corpus of the University of Torino. Moreover, during the intervention period, the project had to sometimes stop its activity due to COVID-19 restrictions, and this fact may have had some influence on participation and thus on the sample’s consistency. Moreover, the female sample was much more consistent than the male sample, even though this result was in line with the reality of the university. Furthermore, the 4-month follow-up period does not represent a long-term monitoring of the maintenance of the good practices, as there was not a third appointment for a subsequent check-up. In addition, since the project was a primary prevention intervention and not a treatment, participants were not supervised during the period between T1 and T2, and as the project was offered to all the employees of the university, we could not form a control group so everyone could freely join the program.

In conclusion, we would like to underline that it is likely probable that the participants who came back for the T2 appointment were those who probably were or became more aware of their actual changes and lifestyle modifications. Despite such issues, more than half of the sample underwent the second assessment, remarking how useful these activities could be for the community.

## 5. Conclusions

Despite such limitations, our promising results showed how a simple and easy-to-apply WHP intervention (using quick and efficient tools like BIVA and Medi-Lite) could help improve worker’s health and some lifestyle habits. The results showed how either body composition or adherence to the Mediterranean diet improved significantly after 4 months. Indeed, such an easy intervention (or similar) could be implemented in other universities, as they represent an optimal environment for the involvement of a wide part of the population, making the interaction between employees and their colleagues possible, who are also the professionals giving them advice for better well-being. Lastly, the good practices achieved are then likely to be spread to families and reach children as well as young adults, who are more and more affected by obesity. Documenting positive examples, even in short-term interventions based on education and dietary correction, in work contexts such as the university one can increase attention and interest towards the topic of correct nutrition and primary health prevention and can be a good example to activate similar projects in other contexts.

## Figures and Tables

**Table 1:** Descriptive statistics of the female sample at T1 and T2 (*n* = 165) and paired *t*-test between T1 and T2 of the female sample.

	Descriptive Statistics				Paired *t*-Test			
	Mean T1	SD T1	Mean T2	SD T2	Delta Mean	Delta DS	*t*	*p*-Value
Age (years)	48.33	9.04						
Height (cm)	161.98	5.92						
Weight (kg)	62.03	9.7	61.63	9.52	−0.4	2.49	−2.07	**<0.01**
BMI (kg/m^2^)	23.66	3.65	23.5	3.5	−0.16	0.93	−2.24	**<0.01**
Waist circumference (cm)	77.65	9.93	75.98	9.55	−1.55	5.11	−3.87	**<0.001**
Hip circumference (cm)	98.72	7.81	97.74	7.69	−0.94	3.85	−3.12	**<0.01**
R/H (ohm/m)	330.33	38.2	326.75	37.7	−3.58	22.79	−2.02	**<0.01**
Xc/H (ohm/m)	32.12	4.77	31.47	4.61	−0.66	3.21	−2.63	**<0.01**
Z/H (ohm/m)	331.91	38.32	328.28	37.81	−3.63	22.91	−2.03	**<0.01**
Rsp (ohm*m)	365.38	60.95	350.36	65.89	−15.02	46.54	−4.15	**<0.001**
Xcsp (ohm*m)	35.51	6.69	33.71	6.86	−1.8	5.09	−4.55	**<0.001**
Zsp (ohm*m)	367.12	61.18	351.99	66.13	−15.13	46.76	−4.16	**<0.001**
PA (°)	5.56	0.63	5.51	0.63	−0.05	0.39	−1.64	0.1
FFM (kg)	46.4	4.1	46.63	4.24	0.23	1.93	1.51	0.1
FFM%	75.78	7.56	76.6	7.58	0.82	3.05	3.47	**<0.001**
FFMI (FFM/m^2^)	17.69	1.45	17.78	1.45	0.09	0.72	1.52	0.1
FM (kg)	15.62	7.11	14.99	6.91	−0.63	2.28	−3.54	**<0.001**
FM%	24.22	7.56	23.4	7.58	−0.82	3.05	−3.47	**<0.001**
FMI (FM/m^2^)	5.96	2.73	5.71	2.64	−0.25	0.88	−3.59	**<0.001**
MM (kg)	29.54	3.27	29.55	3.47	0.01	1.74	0.11	0.5
MM%	48.24	5.88	48.54	5.89	0.3	2.52	1.52	0.1
SMM (kg)	29.54	3.27	29.55	3.47	0.01	1.74	0.11	0.5
ASMM (kg)	17.39	2.16	17.43	2.21	0.04	0.9	0.54	0.3

The bold values indicate the significance.

**Table 2:** Descriptive statistics of the male sample at T1 and T2 (*n* = 81) and paired *t*-test between T1 and T2 of the male sample.

	Descriptive Statistics				Paired *t*-Test			
	Mean T1	SD T1	Mean T2	SD T2	Delta Mean	Delta DS	*t*	*p*-Value
Age (years)	46.54	9.88						
Height (cm)	175.18	7.18						
Weight (kg)	75.5	9.57	75.4	9.69	−0.1	2.54	−0.36	0.36
BMI (kg/m^2^)	24.62	2.88	24.58	2.92	−0.04	0.84	−0.44	0.33
Waist circumference (cm)	88.29	9.37	87.16	9.93	−1.25	5.17	−2.16	**<0.05**
Hip circumference (cm)	99.69	5.9	98.88	5.58	−0.82	3.19	−2.3	**<0.05**
R/H (ohm/m)	257.01	28.15	252.8	27.26	−4.21	18.85	−2.01	**<0.05**
Xc/H (ohm/m)	28.45	4.72	27.81	4.79	−0.64	3.45	−1.68	**<0.05**
Z/H (ohm/m)	258.6	28.35	254.35	27.46	−4.25	19.02	−2.01	**<0.05**
Rsp (ohm*m)	334.64	51.34	319.87	58.45	−14.77	44.14	−3.01	**<0.01**
Xcsp (ohm*m)	37.08	7.49	35.24	8.02	−1.84	5.97	−2.77	**<0.01**
Zsp (ohm*m)	336.72	51.7	321.84	58.83	−14.88	44.44	−3.01	**<0.01**
PA (°)	6.31	0.75	6.27	0.79	−0.04	0.54	−0.7	0.24
FFM (kg)	63.07	6.08	63.6	6.2	0.52	2.7	1.74	**<0.05**
FFM%	84.07	6.45	84.86	6.17	0.79	3.41	2.09	**<0.05**
FFMI (FFM/m^2^)	20.55	1.6	20.73	1.69	0.17	0.89	1.76	**<0.05**
FM (kg)	12.43	6.08	11.81	5.81	−0.62	2.78	−2.01	**<0.05**
FM%	15.93	6.45	15.14	6.17	−0.79	3.41	−2.09	**<0.05**
FMI (FM/m^2^)	4.06	2	3.85	1.89	−0.21	0.92	−2.06	**<0.05**
MM (kg)	42.58	4.7	42.79	4.86	0.21	2.53	0.75	0.23
MM%	56.74	5.34	57.09	5.23	0.35	3.01	1.04	0.15
SMM (kg)	42.58	4.7	42.79	4.86	0.21	2.53	0.75	0.23
ASMM (kg)	25.61	2.69	25.84	2.76	0.22	1.19	1.68	**0.05**

The bold values indicate the significance.

## Data Availability

The raw data supporting the conclusions of this article will be made available by the authors on request.
